# Supplementary material for: Armc5 deletion causes developmental defects and compromises T-cell immune responses
Source: Nat Commun. 2017 Feb 7;8:13834. doi: 10.1038/ncomms13834 (PMC5309699; doi:10.1038/ncomms13834)
Supplement: Supplementary Information — Supplementary figures 1-15 and supplementary table 1. [file ncomms13834-s1.pdf]

Supplementary Figure 1

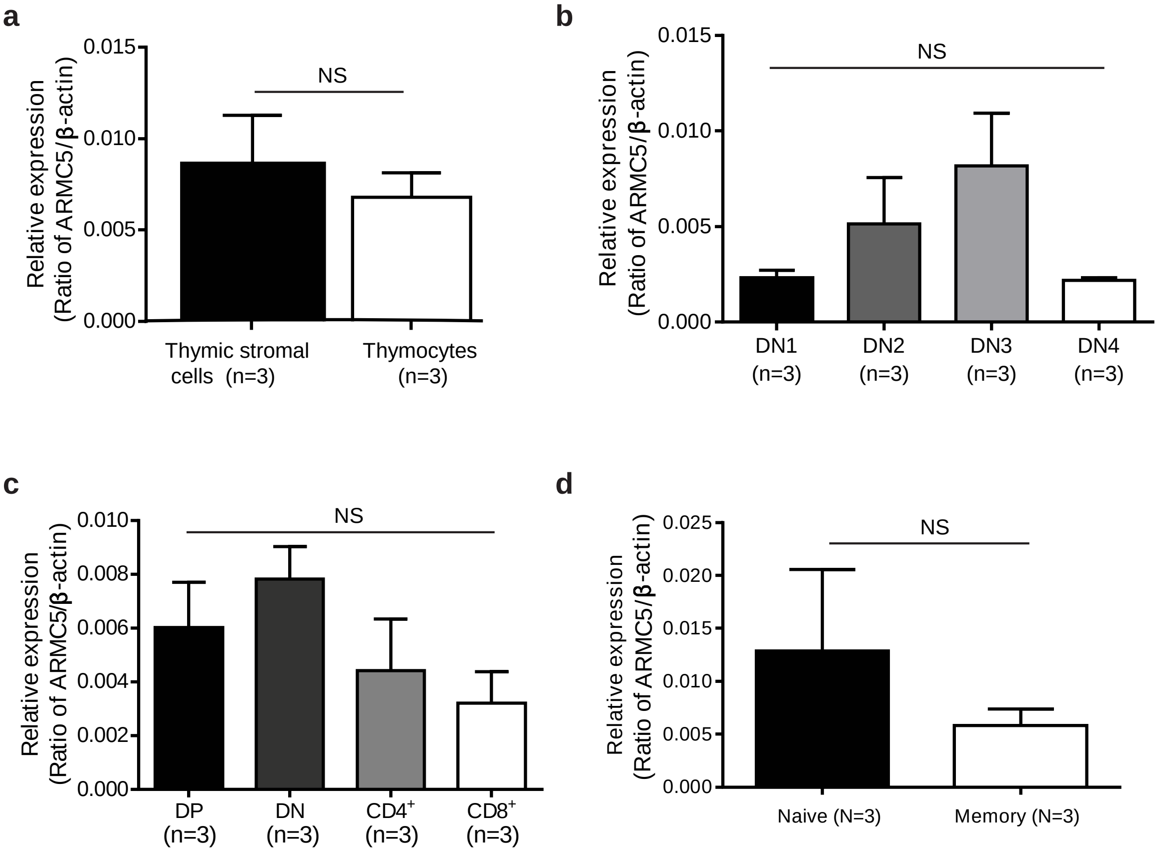

Armc5 mRNA expression in different thymocyte and T-cell subpopulations

RNA was extracted from different subpopulations of thymocytes and T cells, or from T cells cultured under different conditions. *Armc5* mRNA expression levels in these cells were measured by RT-qPCR, with β-actin mRNA levels as internal controls. The numbers (n) of experiments performed are indicated. Pooled results of multiple experiments are expressed as means ± SEM of ratios of *Armc5* versus β-actin signals, unless specified otherwise. Two-tailed Student's *t*-test was used in data between two groups. One way ANOVA followed with Bonferroni's multiple comparisons test was used in data among four groups. NS, no significance.

a. Armc5 mRNA expression in thymocytes and thymic stroma cells

Thymocytes were flushed out from the thymus of WT mice, and the remainder was considered to be thymic stroma cells.

b-c. Armc5 expression in thymocyte subpopulations

Thymocyte subpopulations (B: DN1-4; C: CD4 SP, CD8 SP; CD4CD8 DP, and DN) were sorted by flow cytometry.

d. Armc5 mRNA expression in naïve versus memory T cells

$CD62L^{+}CD44^{lo}$ naïve T cells and $CD62L^{+}CD44^{int\text{-}hi}$ memory T cells were sorted by flow cytometry from WT spleen cells.

Supplementary Figure 2

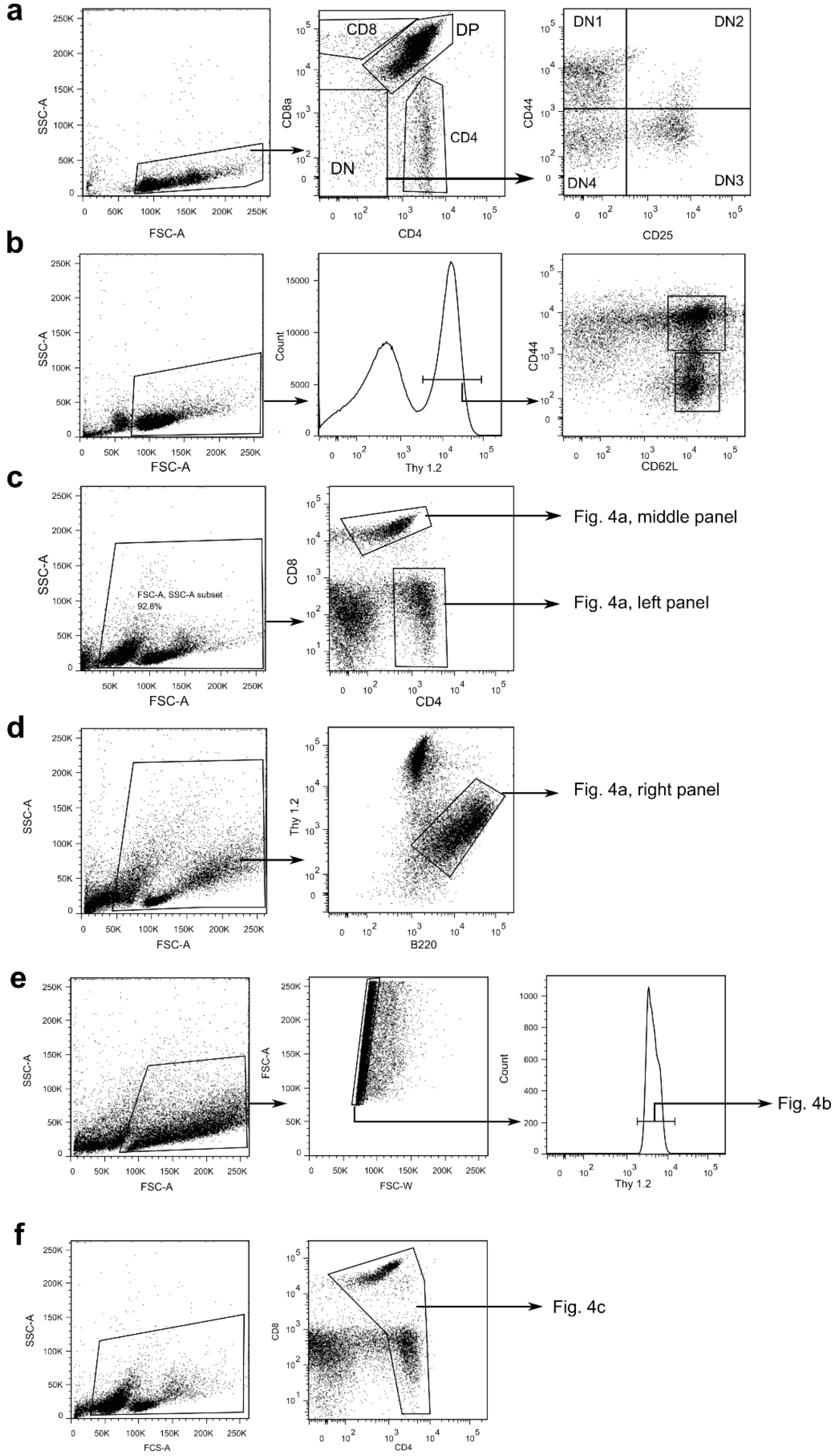

Supplementary Figure 2 continued

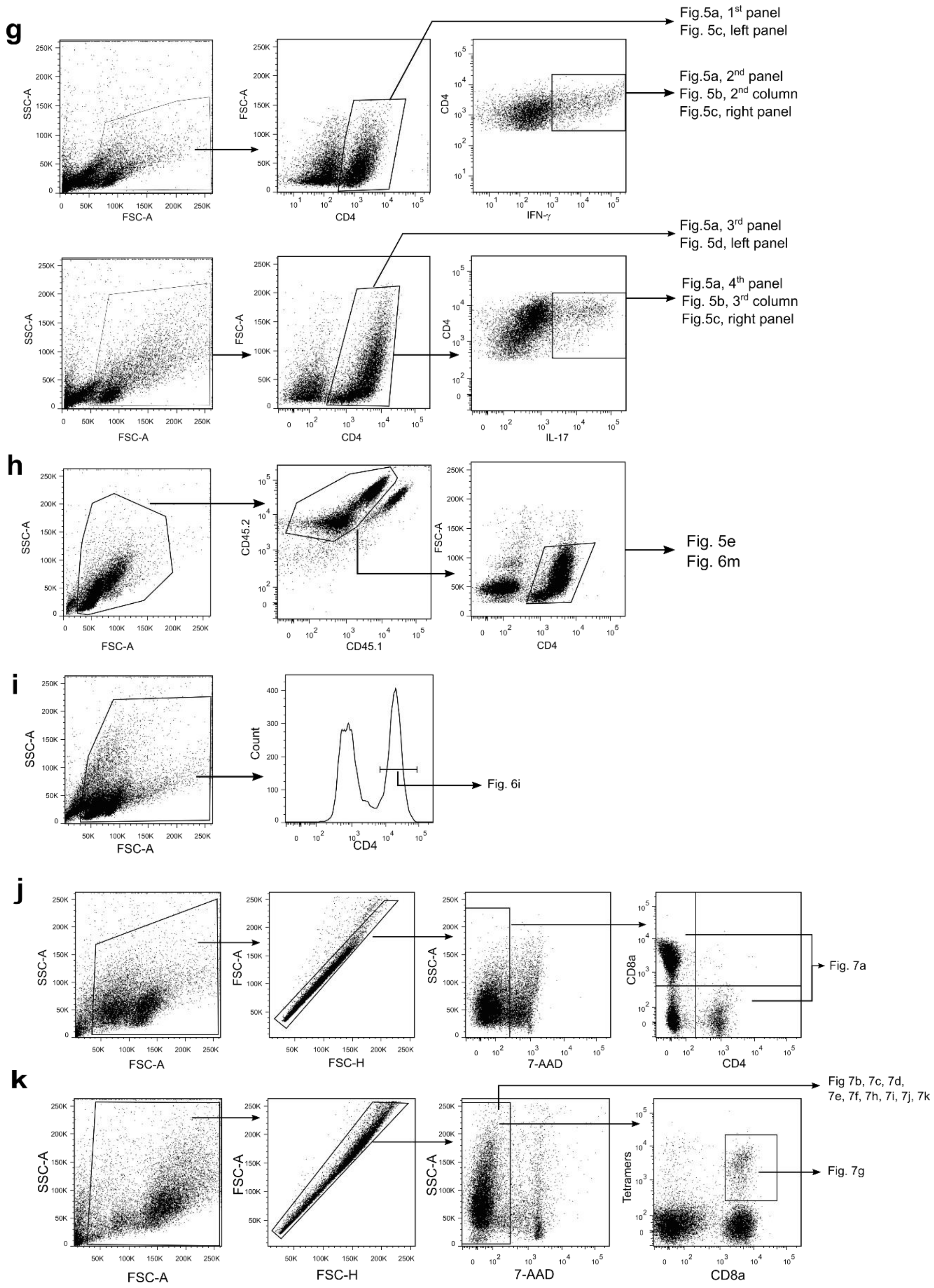

Gating strategies

a. Sequential gating strategy for sorting SP, DP and DN (DN1-4) populations in thymocytes.
b. Sequential gating strategy for sorting memory cells ($CD62L^+CD44^{int\text{-}hi}$) and naïve cells ($CD62L^+CD44^{lo}$) in Thy1.$2^+$ lymph nodes cells.
c. Sequential gating strategy for $CD4^+$ or $CD8^+$ cells in spleen cells.
d. Sequential gating strategy for $B220^+$ cells in spleen cells.
e. Sequential gating strategy for the cell cycle analysis of T cells.
f. Sequential gating strategy for apoptosis analysis of $CD4^+$ and $CD8^+$ cells.
g. Sequential gating strategy for $CD4^+$/$IL17^+$ or $CD4^+$/IFN-γ^+ cells.
h. Sequential gating strategy for $CD4^+$ cells derived from WT and KO donor cells in chimeric mice.
i. Sequential gating strategy for $CD4^+$ cells.
j. Sequential gating strategy for $CD4^+$ or $CD8^+$ cells in LCMV infection mice model.
k. Sequential gating strategy for $CD8^+$ and tetramers$^+$ cells in LCMV infection mice model.

Supplementary Figure 3

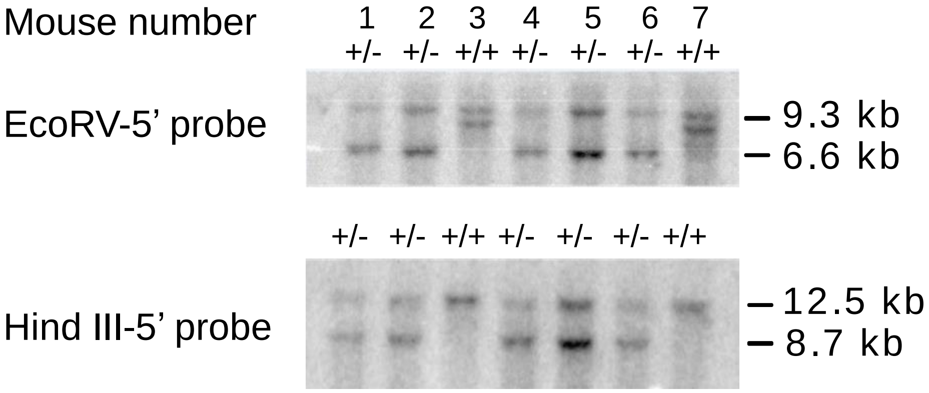

Genotyping of Armc5 mutant mice

Tail DNA was digested with EcoRV and analyzed by Southern blotting (top panel) with the 5’ probe whose location is indicated (Fig. 2a). A 9.3-kb band representing the WT allele and a 6.6-kb band representing the recombinant allele are shown. Similarly, tail DNA was digested with HindIII and analyzed with the 3’ probe (bottom panel). A 12.5-kb band representing the WT allele and an 8.7-kb band representing the recombinant allele are indicated.

Supplementary Figure 4

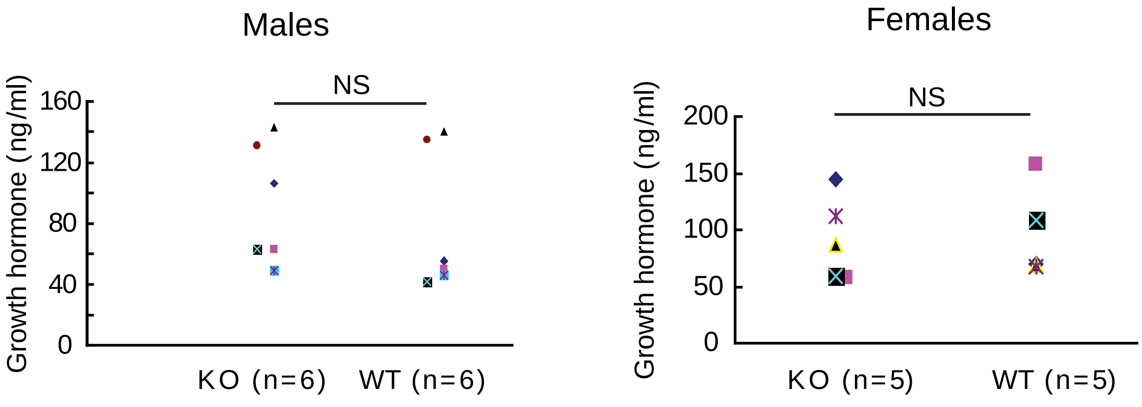

Serum growth hormone levels in KO mice

Serum growth hormone levels in 8-12-week-old KO and WT mice were measured by ELISA. The results are reported as scatter plots, with each symbol representing actual values. Mouse numbers (n) in each group are indicated. p>0.05 (two-tailed Student's t test). NS: not significant.

Supplementary Figure 5

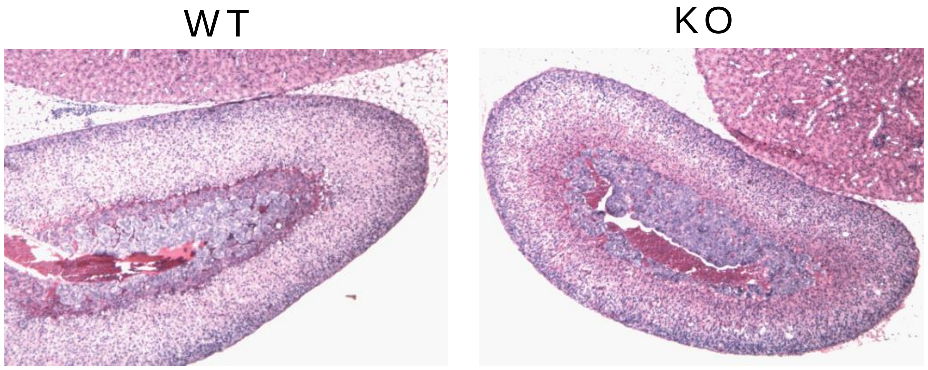

Adrenal gland histology of young KO mice

Adrenal glands from WT and KO mice (8-12 weeks old) were sectioned and stained with H/E. Representative micrographs from a KO (12-week-old male) mouse and its WT male littermate. No histological abnormalities were found in the KO adrenal gland.

Supplementary Figure 6

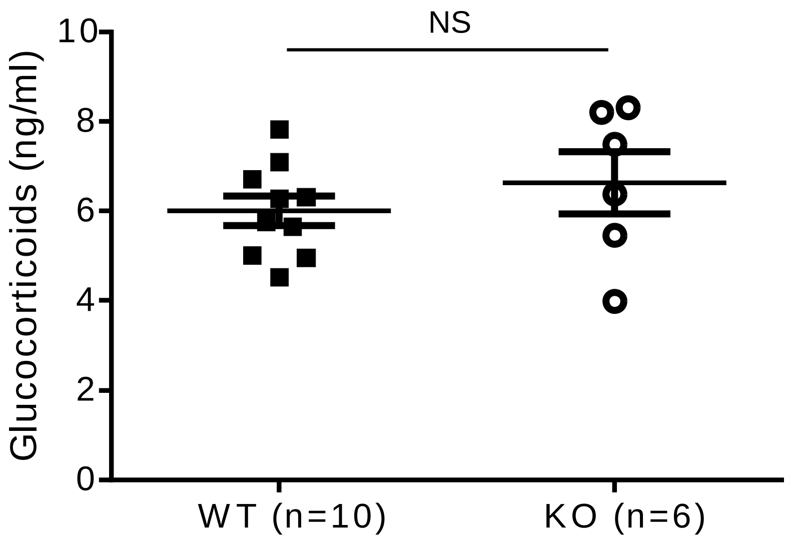

Serum glucocorticoid levels in young WT and KO mice

The mice were bled between 12:30-1:30 pm. Serum levels (means ± SEM) of glucocorticoids old KO and WT mice are shown. Two-tailed Student's *t* test was used for statistical analysis. NS: not significant.

Supplementary Figure 7

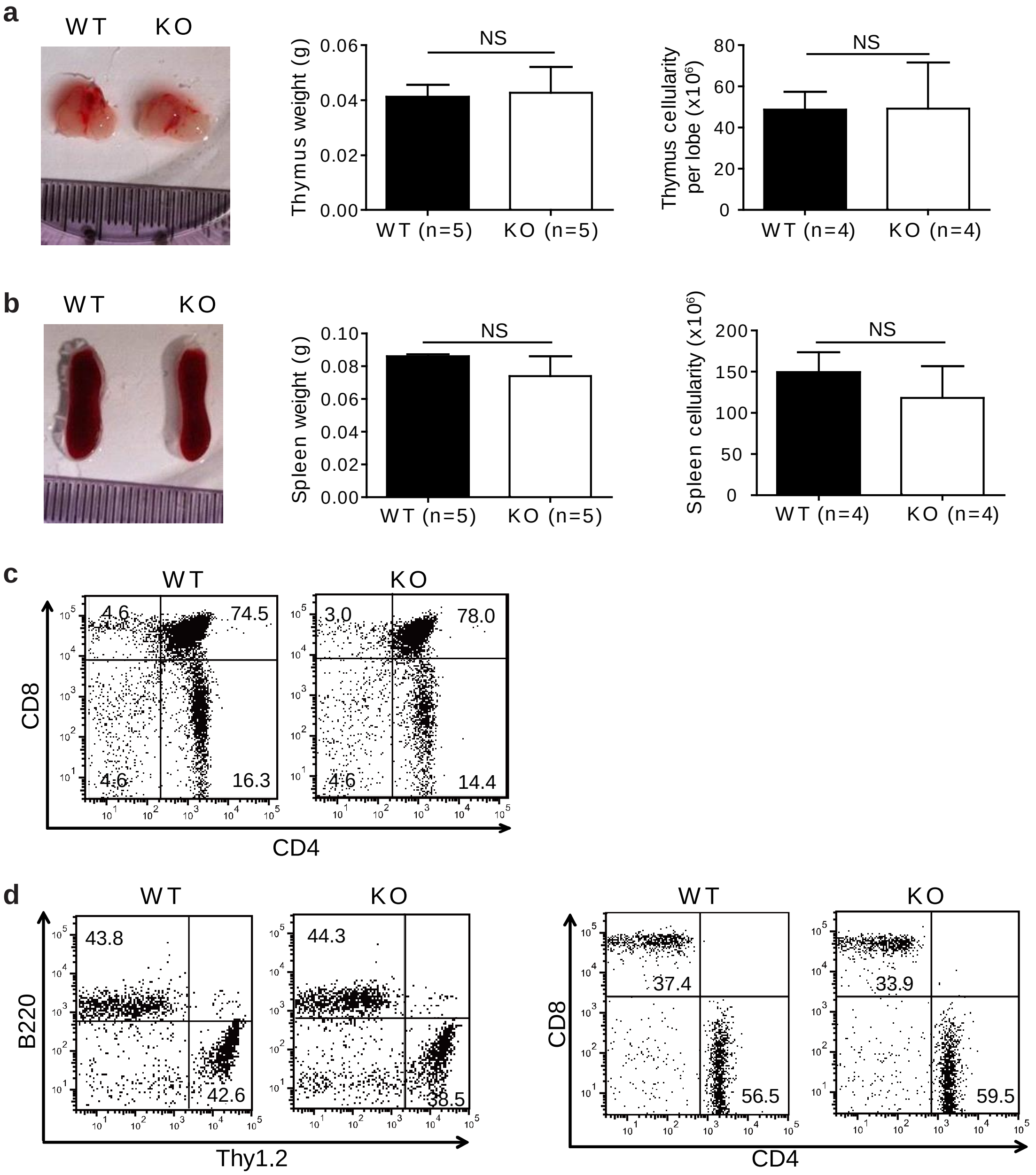

Thymus and spleen weight, cellularity and cell subpopulations in KO mice

a. thymus size, weight, and cellularity in WT and KO mice

Left panel: Representative photo of the KO and WT thymus from 8-week-old littermates. Right panels: thymus weight and cellularity of KO and WT from 8-12-week-old male littermates. Mouse numbers (n) in each group (n) are indicated. p>0.05 (2-tailed Student's t test). NS: not significant.

b. spleen size, weight, and cellularity in WT and KO mice

Left panel: Representative photo of KO and WT spleen from 8-week-old littermates. Right panels: weight and cellularity of KO and WT spleen from 8-12-week-old male littermates. Mouse numbers (n) in each group (n) are indicated. p>0.05 (2-tailed Student's *t* test).

c. T-cell subpopulations in KO thymus in WT and KO mice

Thymocytes from adult KO and WT mice (8-12 weeks old) were analyzed by flow cytometry for percentages of $CD4^+$, $CD8^+$, and $CD4^+CD8^+$ subpopulations. Experiments were conducted more than 3 times. Representative dot plots are reported.

d. Cell subpopulations in WT and KO Spleen

Spleen cells from adult KO and WT mice (8-12 weeks old) were analyzed by flow cytometry for percentages of $Thy1.2^+$ T cells versus $B220^+$ B cells (left panel), and $CD4^+$ versus $CD8^+$ cells (right panel). Experiments were conducted more than 3 times, and representative dot plots are shown.

Supplementary Figure 8

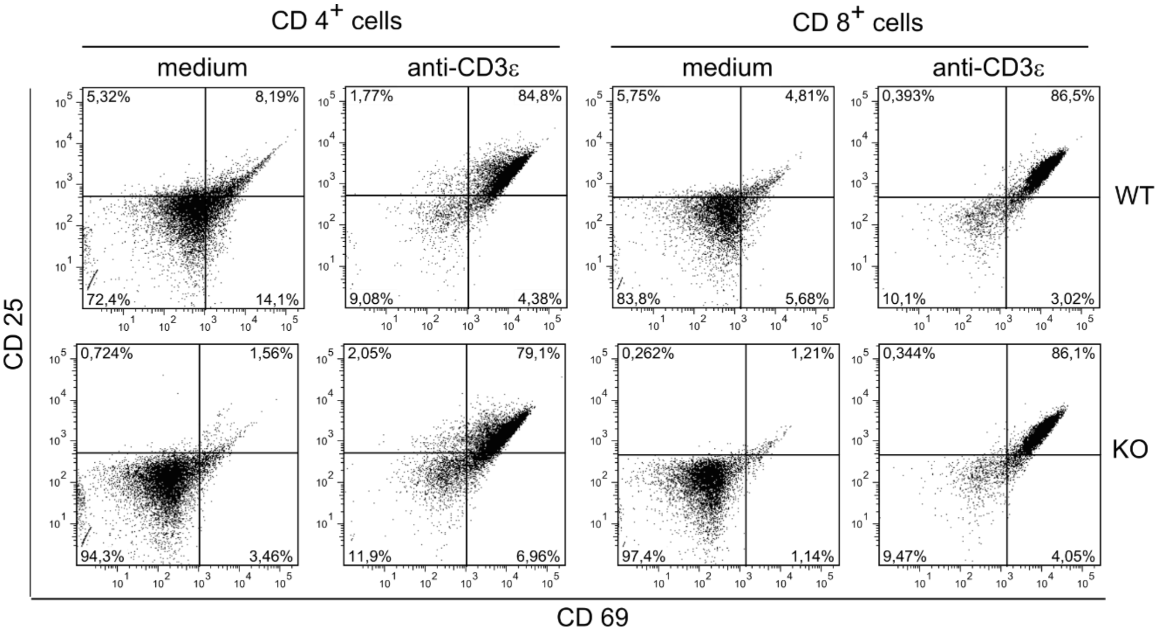

CD25 and CD69 expression in $CD4^+$ and $CD8^+$ cells after anti-CD3ε stimulation

Spleen cells were stimulated with anti-CD3ε mAb(2 μg/ml) for 16 hours. The cells were gated on CD4-positive and CD8-positive. Experiments were conducted independently 3 times. Representative dot plots are shown.

Supplementary Figure 9

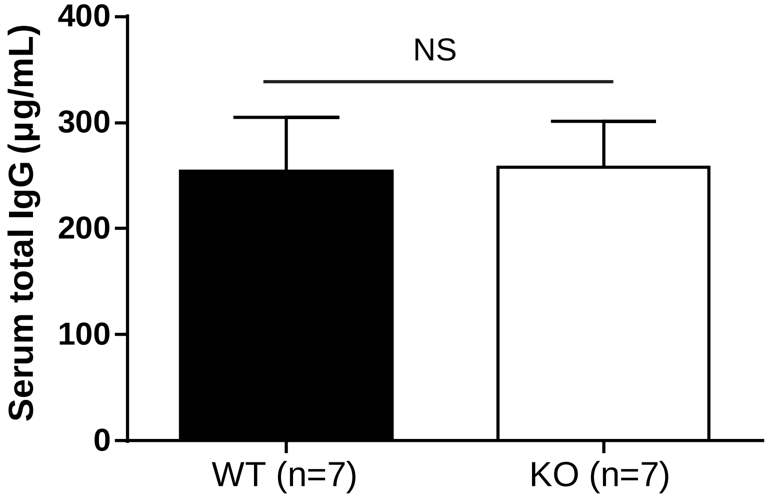

Serum IgG levels in WT and KO mice

Total IgG levels in WT and KO mouse sera were measured by ELISA, and means ± SEM are presented. Mouse numbers (n) in each group are indicated. No statistically significant difference between WT and KO IgG levels was observed (2-tailed Student's *t* test). NS: not significant.

Supplementary Figure 10

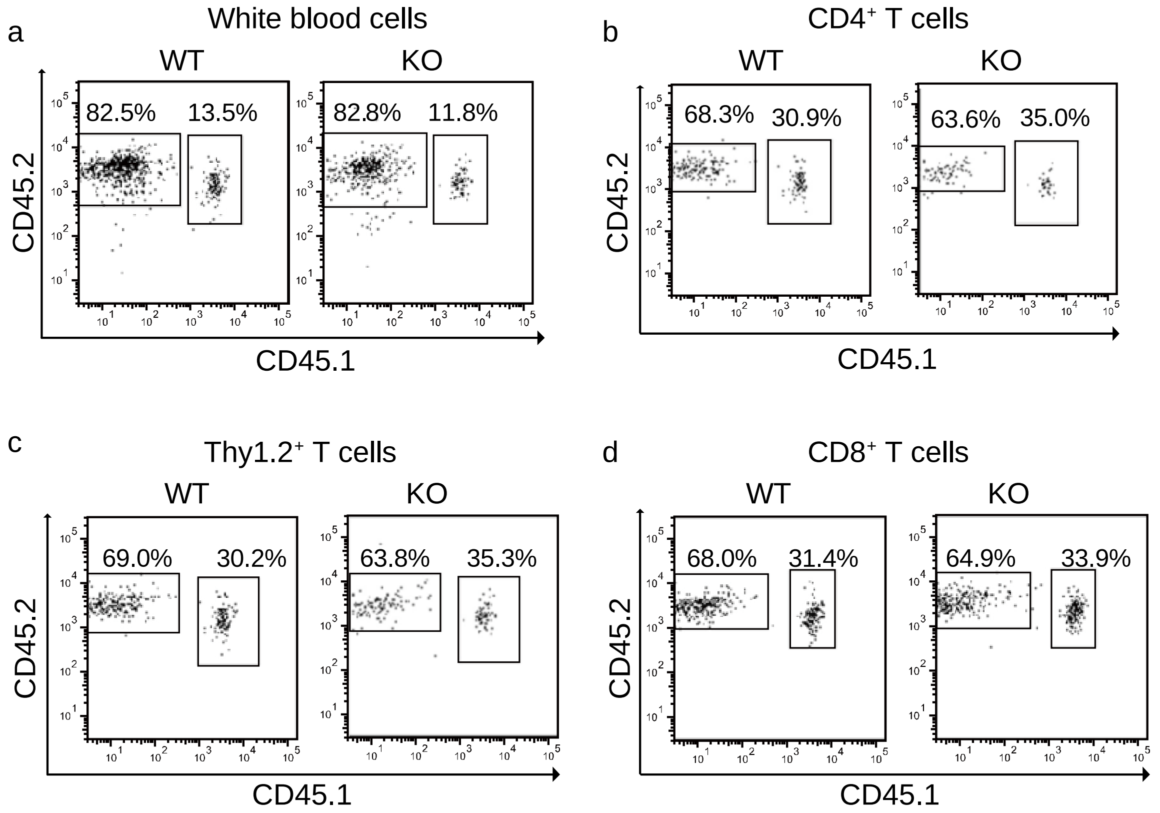

Implantation of donor cells in blood and spleen of chimeric mice

Eight weeks after KO and WT fetal liver transplantation, peripheral blood cells from recipient mice were examined by flow cytometry. Percentages of donor-derived (CD45.2 single-positive cells) versus recipient-derived (CD45.1/CD45.2 double-positive cells; panel a), total T cells (Thy1.2$^+$ cells; panel c), CD4 cells (panel b) and CD8 cells (panel d) in the spleen were measured by flow cytometry. Experiments were conducted more than 4 times, and representative dot plots are shown.

Supplementary Figure 11

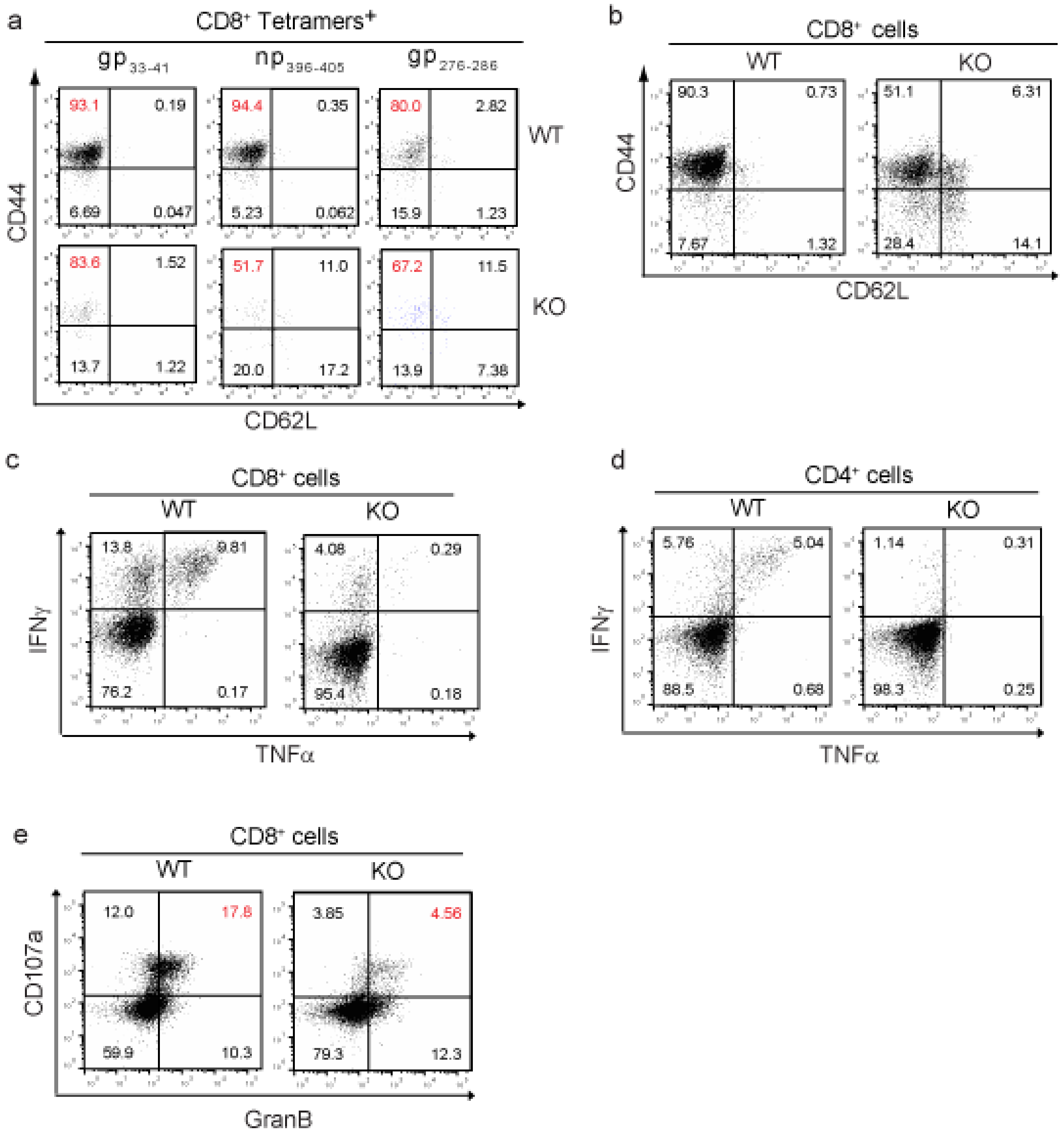

Multiple parameters in spleen T cells on day 8 post-LCMV infection

WT and KO spleen T cells were analyzed for different parameters by flow cytometry on day 8 post-LCMV infection. Representative flow cytometric dot plots are shown (pooled results of the experiments are summarized in bar graphs and presented in Figure 7 in the text proper).

a - b. Activation of LCMV-specific spleen CD8 T cells

On the left panel, gp_{33-41}, $np_{396-405}$ and $gp_{276-286}$ tetramer-positive CD8 cells in the KO and WT mouse spleens were assessed for activation markers ($CD62L^{lo}CD44^{hi}$), and the percentages of this effector memory cell subpopulation are indicated. On the right panel, the dot plot shows the percentages of this population gated on total CD8 cells.

c - d. Expression of IFN-γ and/or TNF-α in gp_{33-41}-stimulated CD8 and gp_{61-80}-stimulated CD4 cells

Dot plots show intracellular IFN-γ and TNF-α expression in WT and KO CD8 (B) and CD4 cells (C) stimulated by gp_{34-41} (for CD8 cells) and gp_{61-80} (for CD4 cells) (both at 5 μM), respectively, for 5 h, in the presence of 50 U/ml IL-2, 5 μg/ml Brefeldin A and 2 μM Monensin.

e. gp_{33-41}-specific $CD107a^+GranB^+$ CD8 T cells on day 8 post-LCMV infection

Spleen cells from KO and WT mice on day 8 post-LCMV infection were stimulated *ex vivo* with gp_{33-41} peptide (5 μM) for 5 h in the presence of 50 U/ml IL-2, 5 μg/ml Brefeldin A, 2 μM Monensin and 2.5 μg/ml FITC-labeled anti mouse CD107a. Percentages of $CD107a^+$ and $GranB^+$ cells among CD8 cells were quantified by flow cytometry.

Supplementary Figure 12

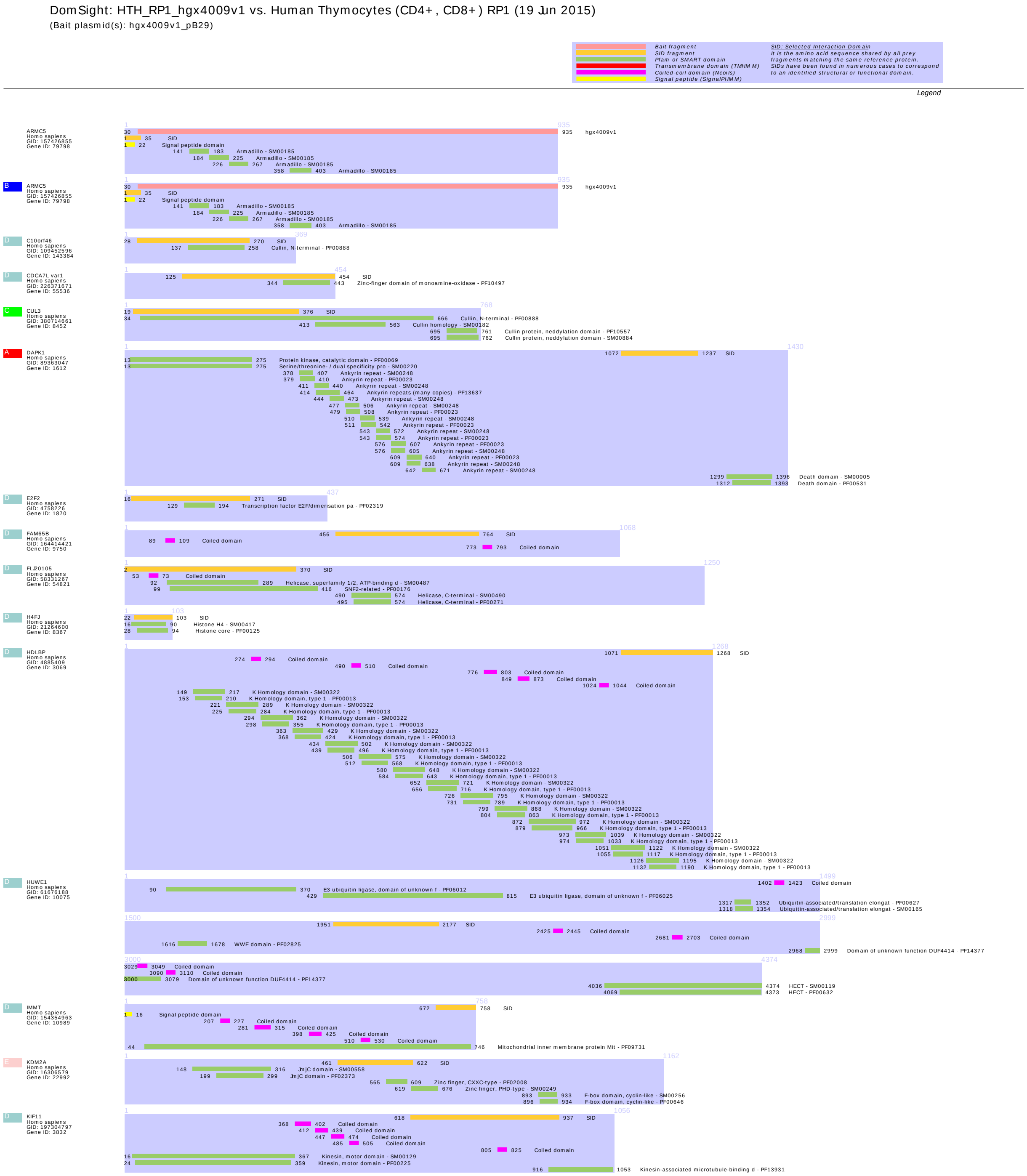

Supplementary Figure 12 continued

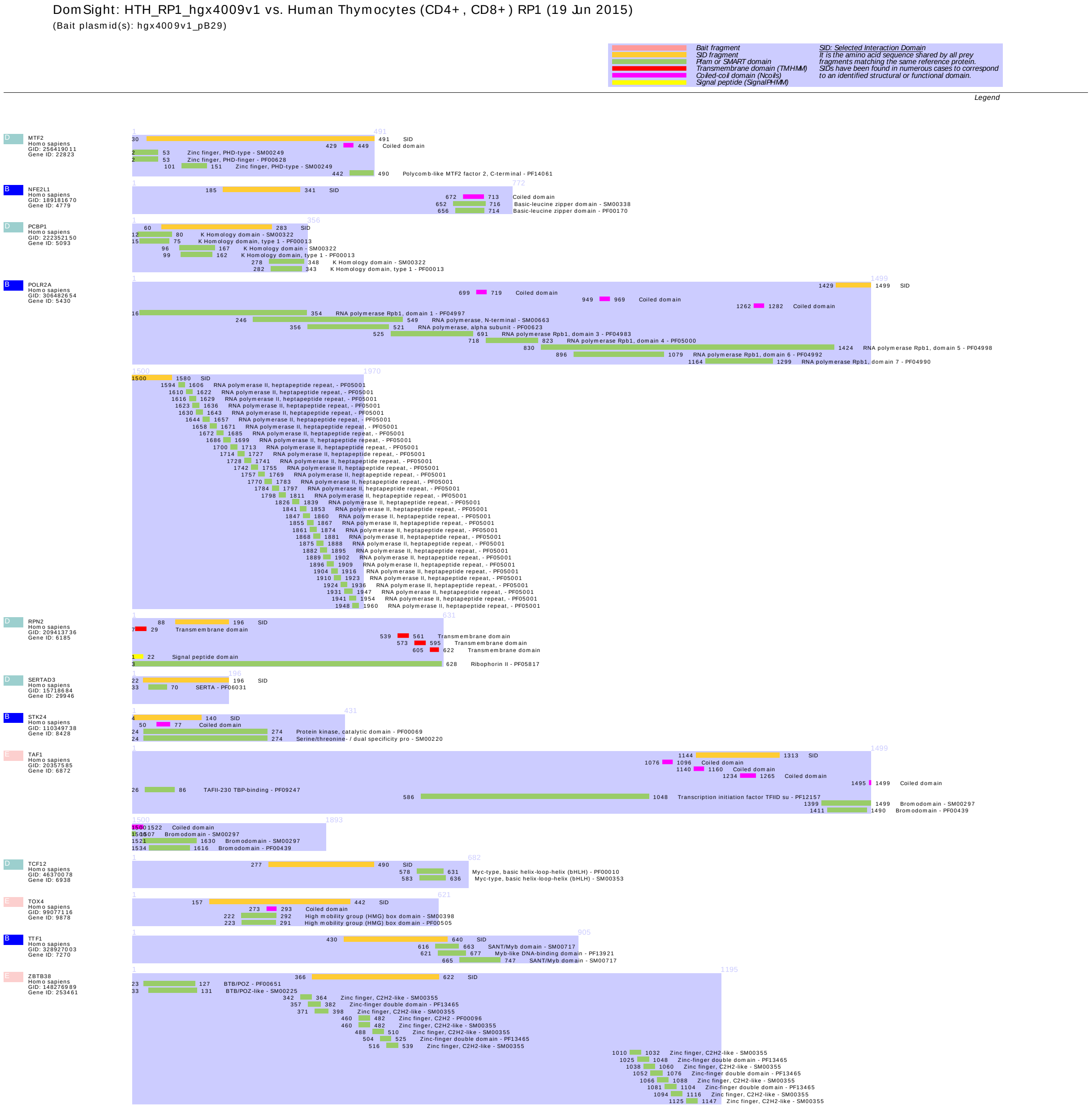

Supplementary Figure 12 continued

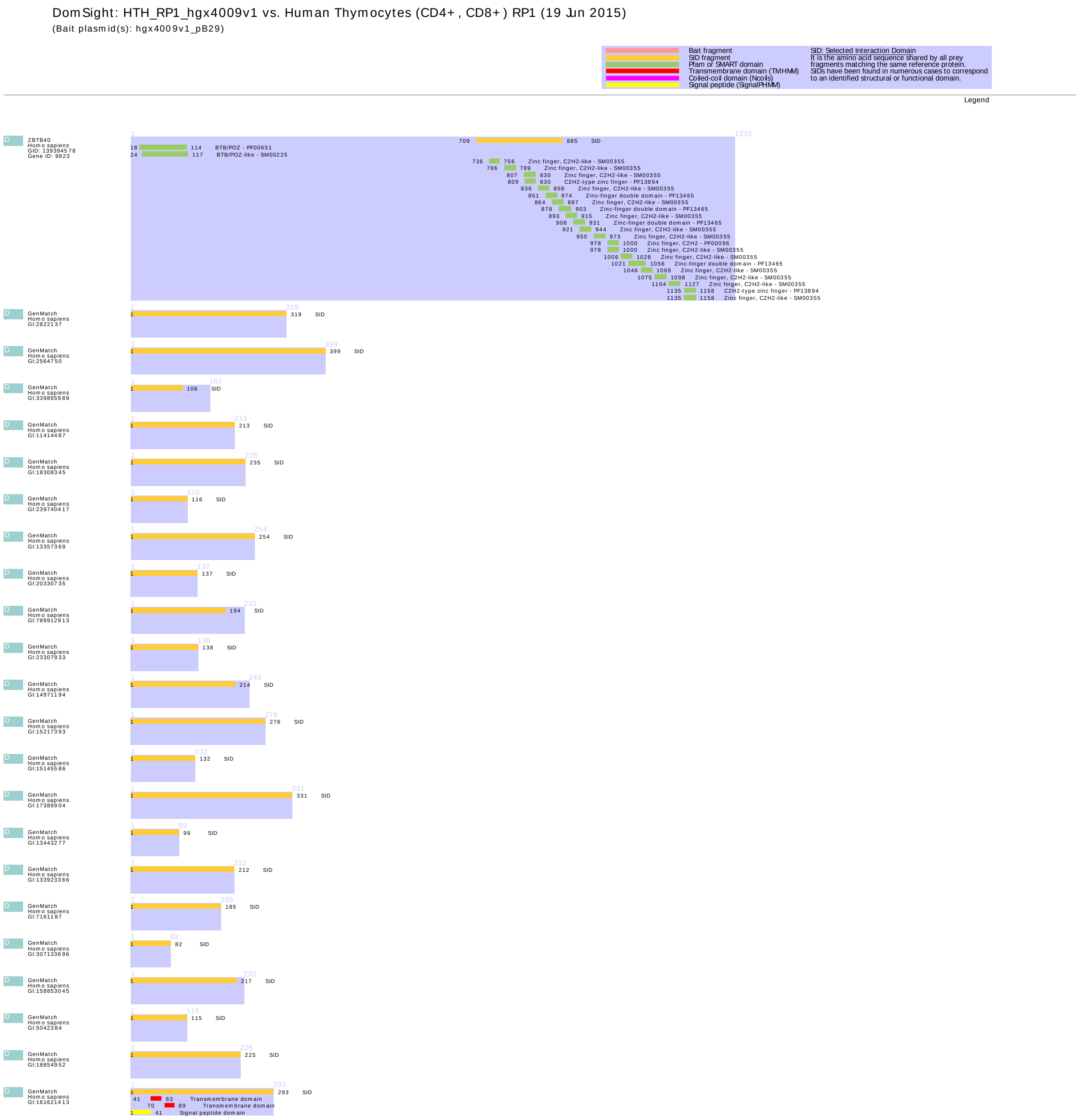

Binding regions between ARMC5 and its associating molecules

The cDNA coding sequences representing the protein binding regions between ARMC5 and its preys are illustrated.

Supplementary Figure 13

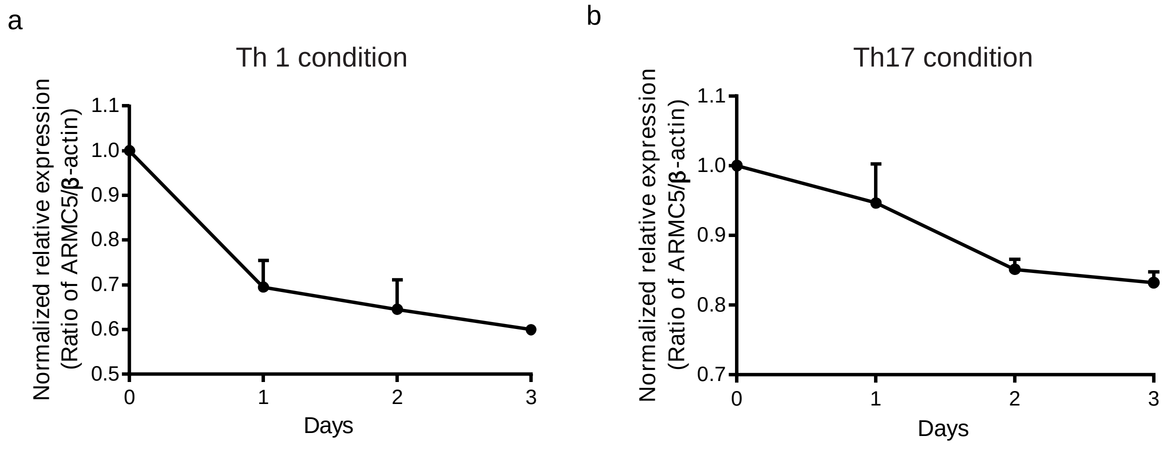

Armc5 mRNA expression in CD4 cells cultured under Th1 and Th17 conditions

WT naïve CD4 cells were cultured under Th1 (panel a) or Th17 (panel b) conditions and harvested at 24, 48 and 72 h. *Armc5* mRNA expression was measured by RT-qPCR. Experiments were conducted more than 3 times and the normalized ratios of *Armc5* versus *β-actin* signals (means ± SEM) of representative experiments are shown. The signal ratios at 0 h are designated as 1.

Supplementary Figure 14

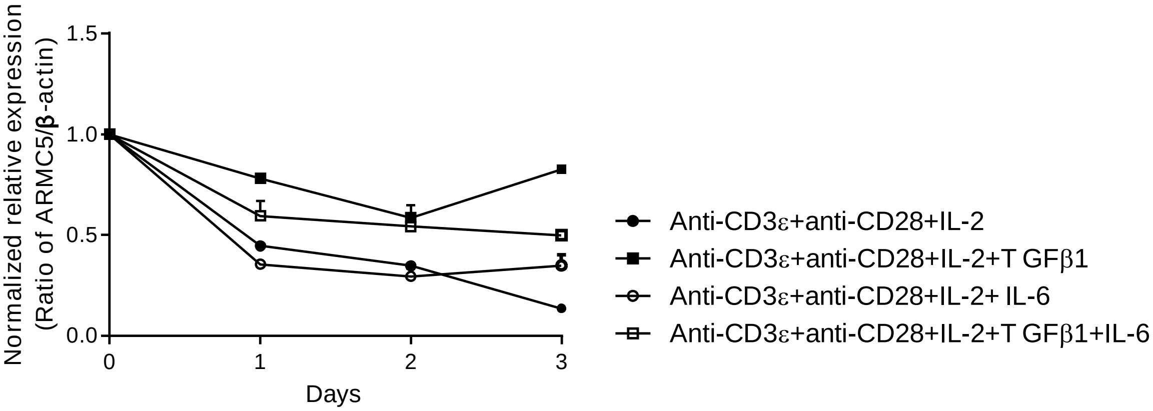

Armc5 mRNA expression in CD4 cells cultured in the presence of different lymphokines

WT naïve CD4 cells were cultured in wells coated with anti-CD3ε and anti-CD28 (0.5μg/ml and 1μg/ml during coating) in the presence of IL-2 (2 μg/ml). In addition, IL-6 (20ng/ml) or TGF-β1 (5 ng/ml), or both was added to culture. The cells were harvested at 24, 48 and 72 h, and their *Armc5* mRNA expression was measured by RT-qPCR. Experiments were conducted more than 3 times and the normalized ratios of *Armc5* versus *β-actin* signals (means ± SEM) of representative experiments are shown. The signal ratios at 0 h are designated as 1.

Supplementary Figure 15

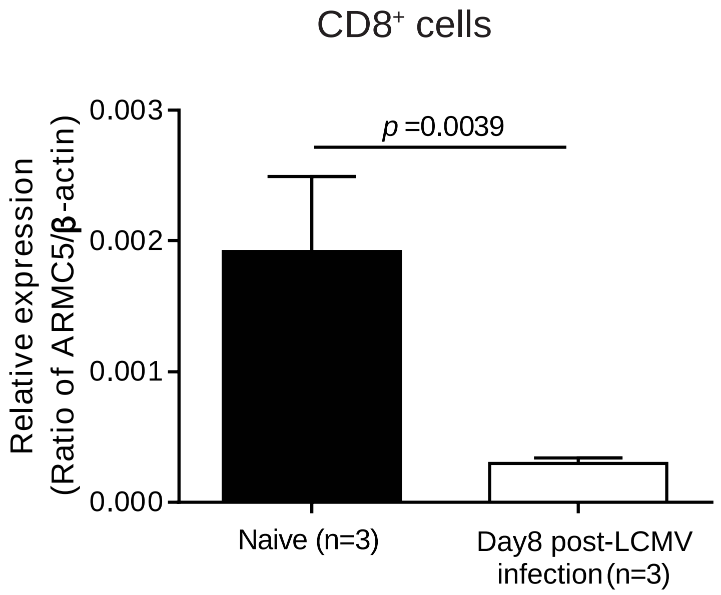

Armc5 mRNA expression in CD8 T cells on day 8 post-LCMV infection

CD8 cells were isolated from the spleens of naïve or LCMV-infected (day 8 post-infection) WT mice, with EasySep™ mouse CD8 T-cell isolation kits. *Armc5* mRNA levels were measured by RT-qPCR. Means ± SEM of ratios of *Armc5* signals versus *β-actin* signals from 3 pairs of mice are shown.

Supplementary Table 1

Summary of adrenal glands hyperplasia in WT and KO mice

	Age (months) Mean ± SD	Bilateral Hyperplasia	Unilateral Hyperplasia
WT (n=5)	20 ± 1.7	0/5*	1/5*
KO (n=5)	18 ± 3.2	3/5*	2/5*

*number of positive mouse/number of total mice
