# Supplementary material for: Armc5 deletion causes developmental defects and compromises T-cell immune responses
Source: Nat Commun. 2017 Feb 7;8:13834. doi: 10.1038/ncomms13834 (PMC5309699; doi:10.1038/ncomms13834)
Supplement: Supplementary Dataset 1 — Results summary of Y2H screen. [file ncomms13834-s2.pdf]

Results Summary

ULTImate Y2H SCREEN
Homo sapiens - ARMC5
# vs Human Thymocytes (CD4+, CD8+) RP1

Fri, Jun 19, 2015 - 09:15 AM

Screen Parameters

Nature	cDNA
Reference Bait Fragment	Homo sapiens - ARMC5 (aa 30-935) ; hgx4009v1
Prey Library	Human Thymocytes (CD4+, CD8+) RP1
Vector(s)	pB29 (N-bait-LexA-C fusion)
Processed Clones	165 (pB29_A)
Analyzed Interactions	80 millions (pB29_A)
3AT Concentration	5.0 mM (pB29_A)

Global PBS®

Global PBS (for Interactions represented in the Screen)		Nb	%
A	Very high confidence in the interaction	1	2.0%
B	High confidence in the interaction	5	10.2%
C	Good confidence in the interaction	1	2.0%
D	Moderate confidence in the interaction<br>This category is the most difficult to interpret because it mixes two classes of interactions :<br>- False-positive interactions<br>- Interactions hardly detectable by the Y2H technique (low representation of the mRNA in the library, prey folding, prey toxicity in yeast)	38	77.6%
E	Interactions involving highly connected (or relatively highly connected) prey domains, warning of non-specific interaction. The total number of screens performed on each organism is taken into account to set this connectivity threshold: 20 interactions to different bait proteins in our entire database for Human, 10 for Mouse, Drosophila and Arabidopsis and 6 for all other organisms. They can be classified in different categories:<br>- Prey proteins that are known to be highly connected due to their biological function<br>- Proteins with a prey interacting domain that contains a known protein interaction motif or a biochemically promiscuous motif	4	8.2%
F	Experimentally proven technical artifacts	0	0.0%
Non Appliable			
N/A	The PBS is a score that is automatically computed through algorithms and cannot be attributed for the following reasons :<br>- All the fragments of the same reference CDS are antisense<br>- The 5p sequence is missing<br>- All the fragments of the same reference CDS are either all OOF1 or all OOF2<br>- All the fragments of the same reference CDS lie in the 5' or 3' UTR		

Prey Fragment Analysis

Symbols	Means
✻	The fragment contains the full length CDS
	Fragment is fully in 5' UTR
	Fragment is fully in 3' UTR
✖	Fragment contains at least one In Frame STOP codon
[NR]	Fragment was found to be non relevant (poor quality, high N density)
IF<br>OOF1<br>OOF2	With regard to the theoretical frame of each corresponding CDS (GeneBank),fragments are cloned in frame (IF) if they are in the same frame as Gal4AD.In general, polypeptides synthesized from OOF fragments are not considered of biological interest, unless found together with another frame. However, some of the proteins expressed from an OOF fragment can be translated in the correct frame,due to the existence of natural frame-shift events during translation in yeast
??	Unidentified frame when :<br>- The clone sequence is antisense<br>- The 5p sequence is missing
N	Antisense
Start...Stop	Position of the 5p and 3p prey fragment ends, relative to the position of the ATG start codon (A=0)

Clone Name	Type Seq	Gene Name (Best Match)	Start..Stop (nt)	Frame	Sense	%Id 5p	%Id 3p	PBS
pB29_A-71	5p/3p	Homo sapiens - APBA2	2251..1808	??	N	100.0	100.0	N/A
pB29_A-44	5p/3p	Homo sapiens - ARMC5	-490..106	IF		99.8	99.7	B
pB29_A-52	5p/3p	Homo sapiens - ARMC5	-388..113	IF		99.0	95.9	B
pB29_A-7	5p/3p	Homo sapiens - BIRC2	519..-186	??	N	87.4	95.5	N/A
pB29_A-67	5p/3p	Homo sapiens - C10orf46	81..811	IF		96.0	91.2	D
pB29_A-142	5p/3p	Homo sapiens - CAMK2D	202..-447	??	N	100.0	98.5	N/A
pB29_A-72	5p/3p	Homo sapiens - CDCA7L var1	373..1380 ✖	IF		100.0	95.7	D
pB29_A-18	5p/3p	Homo sapiens - CEP152	-15..1424	OOF2		99.8	97.2	N/A
pB29_A-138	3p	Homo sapiens - CIZ1	1138..936	??	N		100.0	N/A
pB29_A-39	5p/3p	Homo sapiens - COMMD4	-24..608 ✻ ✖	OOF1		100.0	98.3	N/A
pB29_A-131	5p/3p	Homo sapiens - CUL3	3..1130	IF		97.0	97.2	C
pB29_A-36	5p/3p	Homo sapiens - CUL3	54..1556	IF		99.2	96.7	C
pB29_A-5	5p/3p	Homo sapiens - CYBA	451..-27	??	N	100.0	100.0	N/A
pB29_A-97	5p/3p	Homo sapiens - DAPK1	2958..3804	IF		97.2	97.0	A
pB29_A-132	5p/3p	Homo sapiens - DAPK1	2958..3804	IF		97.5	96.9	A
pB29_A-13	5p/3p	Homo sapiens - DAPK1	2958..3804	IF		95.7	97.0	A
pB29_A-166	5p/3p	Homo sapiens - DAPK1	2958..3804	IF		95.0	94.0	A
pB29_A-20	5p/3p	Homo sapiens - DAPK1	3111..3959	IF		98.0	93.3	A
pB29_A-160	5p/3p	Homo sapiens - DAPK1	3111..3959	IF		96.6	95.5	A
pB29_A-175	5p/3p	Homo sapiens - DAPK1	3192..4035	IF		96.1	95.5	A
pB29_A-73	5p/3p	Homo sapiens - DAPK1	3213..3713	IF		99.8	99.8	A
pB29_A-96	5p/3p	Homo sapiens - DAPK1	3213..3713	IF		99.8	99.8	A
pB29_A-59	5p/3p	Homo sapiens - DAPK1	3213..3713	IF		99.8	99.8	A
pB29_A-181	5p/3p	Homo sapiens - DAPK1	3213..3713	IF		99.8	99.2	A
pB29_A-147	5p/3p	Homo sapiens - DAPK1	3213..3713	IF		98.6	97.1	A
pB29_A-155	5p/3p	Homo sapiens - DAPK1	3213..3713	IF		99.8	99.8	A
pB29_A-24	5p/3p	Homo sapiens - E2F2	45..815	IF		98.2	94.9	D
pB29_A-104	5p	Homo sapiens - EEF1A1	800	OOF2		97.6		N/A
pB29_A-46	5p/3p	Homo sapiens - EIF2AK1	2435..1744	??	N	99.1	96.7	N/A

Clone Name	Type Seq	Gene Name (Best Match)	Start..Stop (nt)	Frame	Sense	%Id 5p	%Id 3p	PBS
pB29_A-107	5p/3p	Homo sapiens - EXT2	-78..241	OOF1		100.0	100.0	N/A
pB29_A-37	5p/3p	Homo sapiens - FAM65B	1365..2293	IF		97.2	95.6	D
pB29_A-137	5p/3p	Homo sapiens - FLJ10706	-155..569 ✗	OOF2		97.2	98.6	N/A
pB29_A-15	5p/3p	Homo sapiens - FLJ20105	3..1110	IF		92.3	94.4	D
pB29_A-64	5p/3p	Homo sapiens - FTSJD2	-150..837	OOF1		97.1	97.3	N/A
pB29_A-75	3p	Homo sapiens - GOLPH3	..523	??			96.2	N/A
pB29_A-9	5p/3p	Homo sapiens - GSE1	2994..2481	??	N	99.8	99.8	N/A
pB29_A-124	5p/3p	Homo sapiens - H4FJ	63..336 ✗	IF		100.0	100.0	D
pB29_A-25	5p/3p	Homo sapiens - HDLBP	3210..3931 ✗	IF		95.6	96.6	D
pB29_A-171	5p/3p	Homo sapiens - HK1	2080..1541	??	N	99.8	100.0	N/A
pB29_A-189	5p/3p	Homo sapiens - HNRNPA2B1 variant B1	652..1275 ✗	OOF1		98.4	86.4	N/A
pB29_A-38	5p/3p	Homo sapiens - HNRNPA2B1 variant B1	703..976	OOF1		100.0	100.0	N/A
pB29_A-6	5p/3p	Homo sapiens - HNRNPA2B1 variant B1	712..1282 ✗	OOF1		100.0	70.3	N/A
pB29_A-91	5p/3p	Homo sapiens - HNRNPA2B1 variant B1	712..1282 ✗	OOF1		100.0	69.8	N/A
pB29_A-78	5p/3p	Homo sapiens - HNRNPA2B1 variant B1	712..1282 ✗	OOF1		100.0	71.4	N/A
pB29_A-90	5p/3p	Homo sapiens - HNRNPA2B1 variant B1	717..1484 ✗	OOF1		99.8	97.1	N/A
pB29_A-116	5p/3p	Homo sapiens - HNRNPA2B1 variant B1	721..1261 ✗	OOF1		100.0	90.1	N/A
pB29_A-92	5p/3p	Homo sapiens - HNRNPA2B1 variant B1	721..1261 ✗	OOF1		100.0	88.4	N/A
pB29_A-186	5p/3p	Homo sapiens - HNRNPA2B1 variant B1	721..1261 ✗	OOF1		100.0	96.0	N/A
pB29_A-19	5p/3p	Homo sapiens - HNRNPA2B1 variant B1	745..1280 ✗	OOF1		100.0	70.9	N/A
pB29_A-84	5p/3p	Homo sapiens - HNRNPA2B1 variant B1	745..1280 ✗	OOF1		100.0	73.0	N/A
pB29_A-110	5p/3p	Homo sapiens - HNRNPA2B1 variant B1	745..1280 ✗	OOF1		99.5	68.2	N/A
pB29_A-54	5p/3p	Homo sapiens - HNRNPA2B1 variant B1	766..1538 ✗	OOF1		96.5	94.2	N/A
pB29_A-183	5p/3p	Homo sapiens - HNRNPA2B1 variant B1	766..1538 ✗	OOF1		96.3	92.9	N/A
pB29_A-30	5p/3p	Homo sapiens - HNRNPA2B1 variant B1	766..1538 ✗	OOF1		97.2	94.1	N/A
pB29_A-35	5p/3p	Homo sapiens - HNRNPA2B1 variant B1	766..1538 ✗	OOF1		97.8	93.7	N/A
pB29_A-32	5p/3p	Homo sapiens - HNRNPA2B1 variant B1	778..1222 ✗	OOF1		100.0	100.0	N/A
pB29_A-79	5p/3p	Homo sapiens - HNRNPA2B1 variant B1	787..1233 ✗	OOF1		81.3	81.3	N/A
pB29_A-11	5p	Homo sapiens - HNRNPA2B1 variant B1	790	OOF1		99.4		N/A
pB29_A-70	5p/3p	Homo sapiens - HNRNPA2B1 variant B1	802..1227 ✗	OOF1		73.1	72.0	N/A
pB29_A-125	5p/3p	Homo sapiens - HNRNPA2B1 variant B1	802..1227 ✗	OOF1		73.1	73.0	N/A
pB29_A-133	5p/3p	Homo sapiens - HNRNPA2B1 variant B1	802..1227 ✗	OOF1		73.1	72.8	N/A
pB29_A-111	3p	Homo sapiens - HUWE1	..6533	??			90.4	D
pB29_A-145	5p/3p	Homo sapiens - HUWE1	5849..6533	IF		99.7	97.7	D
pB29_A-184	5p/3p	Homo sapiens - HUWE1	5849..6533	IF		99.8	96.8	D
pB29_A-108	5p/3p	Homo sapiens - HUWE1	5849..6533	IF		99.8	98.1	D

Clone Name	Type Seq	Gene Name (Best Match)	Start..Stop (nt)	Frame	Sense	%Id 5p	%Id 3p	PBS
pB29_A-48	5p/3p	Homo sapiens - HUWE1	5849..6533	IF		99.8	97.0	D
pB29_A-61	5p/3p	Homo sapiens - HUWE1	5849..6533	IF		99.8	96.9	D
pB29_A-144	5p/3p	Homo sapiens - IMMT	2013..2596 ✗	IF		99.5	99.7	D
pB29_A-33	5p	Homo sapiens - KDM2A	963	IF		97.6		E
pB29_A-121	5p/3p	Homo sapiens - KDM2A	999..2416	IF		97.6	96.9	E
pB29_A-114	5p/3p	Homo sapiens - KDM2A	999..2416	IF		98.6	96.9	E
pB29_A-14	5p/3p	Homo sapiens - KDM2A	1100..1868	IF		99.8	96.0	E
pB29_A-27	5p/3p	Homo sapiens - KDM2A	1100..1868	IF		99.7	95.0	E
pB29_A-28	5p/3p	Homo sapiens - KDM2A	1100..1868	IF		100.0	92.5	E
pB29_A-179	5p/3p	Homo sapiens - KDM2A	1379..2433	IF		100.0	96.5	E
pB29_A-22	5p/3p	Homo sapiens - KDM2A	1379..2433	OOF1		100.0	95.7	E
pB29_A-53	5p/3p	Homo sapiens - KDM2A	1379..2433	IF		100.0	95.2	E
pB29_A-162	5p/3p	Homo sapiens - KIF11	1851..2811	IF		94.0	95.3	D
pB29_A-55	5p/3p	Homo sapiens - MARCH6	753..10	??	N	97.3	97.1	N/A
pB29_A-163	5p	Homo sapiens - MTF2	87	IF		92.7		D
pB29_A-66	5p	Homo sapiens - MTF2	87	IF		96.5		D
pB29_A-29	5p/3p	Homo sapiens - NBEA	410..1181	OOF2		99.8	84.0	N/A
pB29_A-57	5p/3p	Homo sapiens - NDUFS2	1549..1099	??	N	93.0	95.7	N/A
pB29_A-168	5p/3p	Homo sapiens - NFE2L1	551..1120	OOF2		99.6	95.4	B
pB29_A-1	5p/3p	Homo sapiens - NFE2L1	553..1025	OOF1		100.0	98.7	B
pB29_A-158	5p/3p	Homo sapiens - NUP153	2788..3269	OOF1		100.0	100.0	N/A
pB29_A-134	5p/3p	Homo sapiens - OSBPL5	1927..1451	??	N	100.0	100.0	N/A
pB29_A-120	5p/3p	Homo sapiens - OSBPL5	1927..1451	??	N	100.0	100.0	N/A
pB29_A-130	5p/3p	Homo sapiens - OSBPL5	1927..1451	??	N	99.6	99.0	N/A
pB29_A-85	5p/3p	Homo sapiens - PCBP1	177..850	IF		98.8	95.5	D
pB29_A-76	5p/3p	Homo sapiens - PCBP1	177..850	IF		98.4	97.0	D
pB29_A-68	5p	Homo sapiens - PIK3R6	1872..1512	??	N	97.5		N/A
pB29_A-112	3p	Homo sapiens - POLR2A	..4850	??			91.3	B
pB29_A-187	5p/3p	Homo sapiens - POLR2A	4220..4740	OOF2		98.7	99.2	B
pB29_A-109	5p/3p	Homo sapiens - POLR2A	4285..4850	OOF1		100.0	98.3	B
pB29_A-101	5p/3p	Homo sapiens - RAB11FIP3	2389..2080	??	N	99.7	99.7	N/A
pB29_A-93	5p/3p	Homo sapiens - RAB11FIP3	2549..2066	??	N	99.8	96.0	N/A
pB29_A-177	5p/3p	Homo sapiens - RAB11FIP3	2549..2066	??	N	99.8	99.2	N/A
pB29_A-17	5p	Homo sapiens - RAB11FIP3	2549	??	N	94.3		N/A
pB29_A-47	5p/3p	Homo sapiens - RPN2	261..589	IF		100.0	100.0	D
pB29_A-87	5p/3p	Homo sapiens - SERTAD3	62..965 ✗	IF		99.1	95.8	D
pB29_A-140	5p/3p	Homo sapiens - SLC11A2	3250..3658 ✗	OOF1		99.8	99.7	N/A
pB29_A-94	5p/3p	Homo sapiens - SNX14	2888..2131	??	N	98.5	94.4	N/A
pB29_A-113	5p/3p	Homo sapiens - SON	5709..5128	??	N	99.5	99.3	N/A
pB29_A-169	5p/3p	Homo sapiens - SON	5709..5128	??	N	98.8	97.9	N/A
pB29_A-122	5p/3p	Homo sapiens - SON	5709..5128	??	N	99.3	98.6	N/A
pB29_A-173	5p/3p	Homo sapiens - SON	5709..5128	??	N	99.3	99.0	N/A
pB29_A-141	5p/3p	Homo sapiens - SP3	1253..2188	OOF2		98.5	97.2	N/A
pB29_A-148	5p/3p	Homo sapiens - SP3	1253..2188	OOF2		97.5	96.4	N/A
pB29_A-188	5p/3p	Homo sapiens - SP3	1253..2188	OOF2		96.4	95.4	N/A
pB29_A-167	5p/3p	Homo sapiens - SP3	1283..2599 ✗	OOF2		96.9	94.6	N/A
pB29_A-176	5p/3p	Homo sapiens - SP3	1370..2623 ✗	OOF2		97.8	95.9	N/A
pB29_A-69	5p/3p	Homo sapiens - SP4	1190..2190	OOF2		94.5	95.7	N/A
pB29_A-3	5p/3p	Homo sapiens - STK24	-55..420	IF		100.0	100.0	B
pB29_A-63	3p	Homo sapiens - STK24	..584	??			96.0	B

Clone Name	Type Seq	Gene Name (Best Match)	Start..Stop (nt)	Frame	Sense	%Id 5p	%Id 3p	PBS
pB29_A-190	5p	Homo sapiens - STK24	9	IF		94.6		B
pB29_A-146	5p/3p	Homo sapiens - TAF1	3000..4185	IF		97.0	96.9	E
pB29_A-88	5p/3p	Homo sapiens - TAF1	3060..3939	IF		94.8	96.8	E
pB29_A-119	5p/3p	Homo sapiens - TAF1	3060..3939	IF		94.4	95.8	E
pB29_A-2	5p/3p	Homo sapiens - TAF1	3429..4255	IF		96.6	97.9	E
pB29_A-12	5p/3p	Homo sapiens - TAF1	3429..4255	IF		95.2	97.9	E
pB29_A-4	5p/3p	Homo sapiens - TAF1	3429..4255	IF		96.7	97.6	E
pB29_A-62	5p/3p	Homo sapiens - TAF1	3429..4255	IF		96.1	97.0	E
pB29_A-105	5p/3p	Homo sapiens - TAF1	3429..4255	IF		94.4	97.7	E
pB29_A-118	5p	Homo sapiens - TAF1	3429	IF		90.2		E
pB29_A-123	5p/3p	Homo sapiens - TAF1	3429..4255	IF		91.7	100.0	E
pB29_A-152	5p/3p	Homo sapiens - TAF1	3429..4255	IF		95.9	97.8	E
pB29_A-159	5p/3p	Homo sapiens - TAF1	3429..4255	IF		95.7	96.7	E
pB29_A-164	5p/3p	Homo sapiens - TAF1	3429..4255	IF		95.1	96.9	E
pB29_A-170	5p/3p	Homo sapiens - TAF1	3429..4255	IF		92.1	95.8	E
pB29_A-117	5p/3p	Homo sapiens - TCF12	828..1470	IF		98.1	94.7	D
pB29_A-115	5p/3p	Homo sapiens - TOX4	467..1327	IF		99.8	93.4	E
pB29_A-58	5p/3p	Homo sapiens - TTF1	1146..1920	IF		99.4	97.8	B
pB29_A-127	5p/3p	Homo sapiens - TTF1	1287..2331	IF		97.5	96.4	B
pB29_A-31	5p/3p	Homo sapiens - TTF1	1287..2331	IF		96.1	95.1	B
pB29_A-157	5p/3p	Homo sapiens - TUBA1B	43..694	OOF1		99.1	96.3	N/A
pB29_A-45	5p/3p	Homo sapiens - TUBA1B	43..694	OOF1		99.7	94.1	N/A
pB29_A-23	5p/3p	Homo sapiens - TUBB	1179..914	??	N	100.0	100.0	N/A
pB29_A-149	5p/3p	Homo sapiens - WDTC1	1369..2036 ✗	OOF1		99.3	97.2	N/A
pB29_A-26	5p/3p	Homo sapiens - ZBTB38	1095..1866	IF		98.3	95.6	E
pB29_A-106	5p/3p	Homo sapiens - ZBTB40	2124..2655	IF		100.0	100.0	D
pB29_A-89	5p/3p	Homo sapiens - ZBTB40	2124..2655	IF		98.9	92.9	D
pB29_A-172	5p/3p	Homo sapiens - transcription factor Sp1 isoform a	1454..2135	OOF2		98.4	97.5	N/A
pB29_A-86	5p/3p	Homo sapiens - GenMatch GI:2822137	-1..957 ✗	IF		99.6	96.8	D
pB29_A-83	5p/3p	Homo sapiens - GenMatch GI:2564750	-1..1199 ✗	IF		97.5	97.0	D
pB29_A-80	5p/3p	Homo sapiens - GenMatch GI:339895989	-1..319 ✗	IF		100.0	100.0	D
pB29_A-178	5p	Homo sapiens - GenMatch GI:11414487	-1..640 ✗	IF		100.0		D
pB29_A-126	5p/3p	Homo sapiens - GenMatch GI:18308345	-1..1076 ✗	IF		100.0	74.5	D
pB29_A-51	5p/3p	Homo sapiens - GenMatch GI:239740417	-2..348 ✗	IF		100.0	100.0	D
pB29_A-81	5p	Homo sapiens - GenMatch GI:13357369	-1	IF		100.0		D
pB29_A-49	5p/3p	Homo sapiens - GenMatch GI:20330735	-1..413 ✗	IF		99.8	97.8	D
pB29_A-43	5p/3p	Homo sapiens - GenMatch GI:789912913	-1..583 ✗	IF		100.0	100.0	D
pB29_A-65	5p	Homo sapiens - GenMatch GI:23307933	-2	IF		100.0		D
pB29_A-56	5p/3p	Homo sapiens - GenMatch GI:14971194	-1..642 ✗	IF		100.0	96.4	D
pB29_A-182	5p/3p	Homo sapiens - GenMatch GI:15217393	-1..828 ✗	IF		97.6	94.4	D
pB29_A-180	5p/3p	Homo sapiens - GenMatch GI:15145586	-1..397 ✗	IF		100.0	100.0	D

Clone Name	Type Seq	Gene Name (Best Match)	Start..Stop (nt)		Frame	Sense	%Id 5p	%Id 3p	PBS
pB29_A-174	5p/3p	Homo sapiens - GenMatch GI:17389904	-1..995	✗	IF		96.6	95.9	D
pB29_A-161	5p/3p	Homo sapiens - GenMatch GI:13443277	-1..298	✗	IF		100.0	100.0	D
pB29_A-154	5p/3p	Homo sapiens - GenMatch GI:133923366	-1..636	✗	IF		99.5	99.7	D
pB29_A-151	5p/3p	Homo sapiens - GenMatch GI:7161187	-1..556	✗	IF		99.1	100.0	D
pB29_A-129	5p/3p	Homo sapiens - GenMatch GI:307133696	-1..246	✗	IF		100.0	100.0	D
pB29_A-128	5p/3p	Homo sapiens - GenMatch GI:158853045	-1..653	✗	IF		99.6	98.8	D
pB29_A-10	5p/3p	Homo sapiens - GenMatch GI:5042384	-1..346	✗	IF		99.7	100.0	D
pB29_A-102	5p/3p	Homo sapiens - GenMatch GI:18854952	-1..675	✗	IF		99.4	99.6	D
pB29_A-95	5p/3p	Homo sapiens - GenMatch GI:161621413	-1..879	✗	IF		92.3	93.5	D
